# Novel Umami Peptides from *Hypsizygus marmoreus* and Interaction with Umami Receptor T1R1/T1R3

**DOI:** 10.3390/foods12040703

**Published:** 2023-02-06

**Authors:** Xiaobo Dong, Chao Wan, Aiyun Huang, Huaide Xu, Hongjie Lei

**Affiliations:** College of Food Science and Engineering, Northwest A&F University, Xianyang 712100, China

**Keywords:** *Hypsizygus marmoreus*, umami peptide, molecular docking, molecular dynamics simulation

## Abstract

Umami peptides are important taste components of foods. In this study, umami peptides from *Hypsizygus marmoreus* hydrolysate were purified through ultrafiltration, gel filtration chromatography, and RP-HPLC, and then identified using LC-MS/MS. The binding mechanism of umami peptides with the receptor, T1R1/T1R3, was investigated using computational simulations. Five novel umami peptides were obtained: VYPFPGPL, YIHGGS, SGSLGGGSG, SGLAEGSG, and VEAGP. Molecular docking results demonstrated that all five umami peptides could enter the active pocket in T1R1; Arg277, Tyr220, and Glu301 were key binding sites; and hydrogen bonding and hydrophobic interaction were critical interaction forces. VL-8 had the highest affinity for T1R3. Molecular dynamics simulations demonstrated that VYPFPGPL (VL-8) could be steadily packed inside the binding pocket of T1R1 and the electrostatic interaction was the dominant driving force of the complex (VL-8-T1R1/T1R3) formation. Arg residues (151, 277, 307, and 365) were important contributors to binding affinities. These findings provide valuable insights for the development of umami peptides in edible mushrooms.

## 1. Introduction

Umami, described as a delicious or pleasant savory taste, is the fifth basic taste. Umami taste is a critical factor in food flavor. A variety of components has been reported to elicit umami taste, including some bi-functional acids, free amino acid, and peptides and their derivatives [1,2,3]. Umami substances have been found in many foods, including seafood, meat, and vegetables, especially in edible mushrooms [4,5]. It is well known that the most prominent flavor feature of edible mushrooms is their umami taste. The umami taste of mushrooms mainly results from specific amino acids, nucleotides, and peptides [6,7]. *Hypsizygus marmoreus*, also known as the “seafood mushroom” due to its delicious taste, is popular with consumers, especially those in Asia. Previous researchers have investigated the umami taste of *H. marmoreus*, including umami 5′-nucleotides and amino acids [8]. However, the umami peptides in *H. marmoreus* have not been investigated.

Umami peptides have attracted increasing attention owing to their good palatability, complex taste, as well as biological functions [5,9,10]. Umami peptides are mainly derived from food sources with high protein, such as meat, soybeans, marine products, and mushrooms [11,12,13]. *H. marmoreus*, rich in protein and umami amino acids, is an excellent potential source of umami peptides [8]. Since the discovery of the typical peptide, Lys-Gly-Asp-Glu-Glu-Ser-Leu-Ala, from beef hydrolysate, a growing number of umami peptides has been identified, including Gly-Glu, Glu-Pro-Glu, Ser-Glu-Glu-Lys, and Asp-Ala-Leu-Lys-Lys-Lys [11,13,14]. Umami taste is initiated through the binding of umami components to umami receptors. To date, various G protein-coupled receptors (GPCRs) responsible for umami substance recognition have been reported, including T1R1/T1R3, mGluRs, and CaSR [15,16,17]. Among these, T1R1/T1R3 has been proven to be the primary receptor for umami taste [18]. T1R1/T1R3 can be activated through the binding of umami peptides to the pocket of the extracellular Venus flytrap domain (VFTD). The binding modes can be very different for distinct umami peptides. Compared with short-chain umami peptides, the interaction between long-chain peptides and receptors is more complicated due to their complex amino acid composition and spatial structure [5,17]. *H. marmoreus* hydrolysate contains potential umami peptides, and the interaction involved between diverse umami peptides and T1R1/T1R3 might be different. However, the binding mechanism of umami peptides in *H. marmoreus* and receptors remains unclear [8,19].

Therefore, the objective of this study was to purify and identify the potential umami peptides in *H. marmoreus* and reveal the interaction between umami peptides and T1R1/T1R3. This study will provide useful information for the development of natural edible mushroom umami enhancers.

## 2. Materials and Methods

### 2.1. Materials and Chemicals

*H. marmoreus* was purchased from Wuhan Yuanlai Ecological Agricultural Technology Co., Ltd. (Wuhan, China). Flavourzyme (30 U/mg, from *Aspergillus oryzae*), protamex (100 U/mg, from *Hay bacillus*), and MSG were obtained from Solarbio (Beijing, China). Sephadex G-15 was obtained from GE Healthcare (Uppsala, Sweden). Trifluoroacetic acid (TFA) and acetonitrile (ACN) were acquired from Fisher Scientific (Shanghai, China). All other chemicals and reagents were of analytical grade.

### 2.2. Preparation of H. marmoreus Hydrolysate

*H. marmoreus* hydrolysate was prepared according to our previous study [19]. Fresh mushrooms were dried using an electric incubator (GZX-9240MBE, Yuhua Equipment Ltd., Gongyi, China) at 60 °C, until the moisture content was reduced below 10% wet basis, then ground and filtrated through 60 mesh. Stipe powder was added to deionized water at a ratio of solid to water (*w*/*v*) of 1:15, and the pH was adjusted to 6.5. The suspension was hydrolyzed with protease of 4000 U/g (flavourzyme: protamex was 1:2) at 50 °C for 2 h, and then incubated at 100 °C for 10 min to inactivate the enzymes. The hydrolysate was centrifuged at 8000× *g* for 5 min at 4 °C, and the supernatant was then collected. The hydrolysate was frozen in an ultra-low temperature refrigerator at −80 °C in advance, and then lyophilized in the freeze dryer at −50 °C with a vacuum degree less than 10 Pa.

### 2.3. Separation and Purification of Umami Peptides

#### 2.3.1. Ultrafiltration of Hydrolysate

Ultrafiltration was carried out according to Kong et al. (2019) [14]. The hydrolysate was then ultrafiltered using membranes (Millipore, Bedford, MA, USA) with two molecular weight (MW) cutoffs (3 kDa and 1 kDa) at 4 °C. The fractions U-I (>3 kDa), U-II (1–3 kDa), and U-III (<1 kDa), respectively, were collected and then lyophilized for umami evaluation.

#### 2.3.2. Purification of Umami Peptides by Gel Filtration Chromatography (GFC)

GFC was conducted according to Kong et al. (2019) [14]. The most intense umami taste fraction isolated from ultrafiltration (50 mg/mL) was eluted on a Sephadex G-15 GFC column (1.6 × 70 cm) with deionized water at a flow rate of 1 mL/min (3 mL per tube). The absorbance of the elution was detected at 220 nm. The resulting fractions were collected and lyophilized for further evaluation.

#### 2.3.3. Purification of Umami Peptides by RP-HPLC

The most intense umami fraction obtained through GFC was further subjected to an RP-HPLC system (CBM-20A, Shimadzu, Japan) according to Kong et al. (2019) [14], with some modifications. The fraction (50 mg/mL, 0.2 mL) was loaded onto a Shim-pack GIST-HP C18 column (150 mm × 10 mm, 3 μm, Shimadzu, Japan). The mobile phase was ultrapure water containing 0.05% (*v*/*v*) TFA (A) and CAN containing 0.05% (*v*/*v*) TFA (B). The gradient elution was set as 0–15 min, 90–50% A; 15–25 min, 50–10% A; 25–40 min, 10–90% A, at a flow rate of 1 mL/min. The detector wavelength was monitored at 214 nm. The resulting fractions were collected and lyophilized for further evaluation.

### 2.4. Sensory Evaluation

The sensory evaluation was conducted as described by Li et al. (2020) [20], with some modifications. Ten sensory panelists (5 males and 5 females aged 24–28) were recruited. All sensory panelists had no taste or smell disorders. They were well trained to recognize the five basic tastes, and had at least one year of sensory evaluation experience. Taste intensity was expressed using a 10-point scale, with 0 points meaning no taste and 10 points meaning strong taste. Five standard solutions (5 points), including citric acid (0.08%, *w*/*v*), sucrose (1%, *w*/*v*), MSG (0.35%, *w*/*v*), L-isoleucine (0.25%, *w*/*v*), and NaCl (0.35%, *w*/*v*) solutions, were employed to evaluate sour, sweet, umami, bitter, and salty tastes, respectively. Aqueous solutions containing 10 mg/mL ultrafiltration fraction, 1 mg/mL GFC fraction, and 0.1 mg/mL RP-HPLC fraction, respectively, were prepared for evaluation. In order to avoid the effects of fatigue and residual solutions, panelists were required to rinse twice with 50–60 mL of drinking water and rest for two minutes between two evaluations.

### 2.5. Electronic Tongue Measurement

The α-ASTREE electronic tongue system (Alpha M.O.S. Co., Toulouse, France), equipped with 7 sensors (ZZ, BB, JE, HA, CA, GA, and JB), was employed to determine the taste properties of samples according to Dang et al. (2019) and Kong et al. (2019), with minor modifications [14,21]. MSG solution (0.35%) was employed as the umami taste reference. Aqueous solutions containing 10 mg/mL ultrafiltration fraction, 1 mg/mL GFC fraction, and 0.1 mg/mL RP-HPLC fraction, respectively, were prepared for evaluation. Each parallel measurement was repeated 7 times, and the last three repeated data were selected for Principal Component Analysis (PCA).

### 2.6. Identification of Umami Peptides Using Nano LC-MS/MS

The most intense umami peptides from the RP-HPLC fraction were identified using a Nano LC-MS system with a Q Exactive™ Hybrid Quadrupole-Orbitrap™ Mass Spectrometer equipped with an Easy-nLC1200 UPLC system (ThermoFisher Scientific, USA) [22]. The UPLC chromatographic conditions were as follows: C18 column (ReproSil-Pur C18-AQ resin column, 150 μm × 15 cm, 1.9 μm, Dr. Maisch GmbH, Germany); mobile phase A was 0.1% formic acid in water, B was 20% 0.1% formic acid in water-80% acetonitrile; flow rate was 600 nL/min. The gradient elution program was as follows: 0–2 min, 96–92% A; 2–45 min, 92–72% A; 45–55 min, 72–60% A; 55–56 min, 60–5%; 55–66 min, 5%. Mass spectrometry conditions were as follows: spray voltage was 2.2 kV; capillary temperature was 270 °C. The primary mass spectrum scan mode was 300–1600 m/z, MS resolution was 60,000, separation width was 3.00 Da. Ten ions with the highest ionic strength in the primary mass spectrometry were selected for MS/MS spectrometric dissociation.

### 2.7. Homology Modeling and Molecular Docking

The 3D structure of T1R1/T1R3 was constructed using homology modeling according to Liu et al. (2019) and Wang et al. (2022) [23,24]. The fish taste receptor T1R2a-T1R3 (PDB ID:5X2M) was employed as a template to construct the model of T1R1-T1R3 using the SWISS-MODEL. Molecular dynamics simulation was carried out to optimize the model. The 3D structure of peptides was drawn in Chemoffice, then optimized using MM2. Molecular docking was performed with Auto Dock Vina according to Liang et al. (2022) [25].

### 2.8. Molecular Dynamics Simulation Studies

Molecular dynamics simulations were conducted with Gromacs 2019.5 according to Liang et al. (2021) [25]. Molecular dynamics simulation of umami peptides and the peptide T1R1/T1R3 complex were performed for 50 ns. The binding free energies (Δ*G*_bind_) of the umami peptides and T1R1/T1R3 were estimated using the MM-PBSA method [26].

### 2.9. Statistical Analysis

The experiments were conducted in triplicate and the results were expressed as mean values ± standard deviation. The differences in mean values were compared by conducting Duncan’s multiple tests using SPSS 18.0, and the level of significance was set at *p* < 0.05.

## 3. Results and Discussion

### 3.1. Purification of Umami Peptides

The potential umami peptides were purified from *H. marmoreus* hydrolysate using ultrafiltration, GFC, and RP-HPLC. Sensory evaluation has been widely used to directly describe the umami taste of various peptides [13,20,27]. The electronic tongue can simulate the human tongue concept and obtain a comprehensive assessment of taste characteristics [14,28]. Thus, the umami taste of isolated fractions was determined through sensory evaluation combined with the electronic tongue.

**Figure 1:**
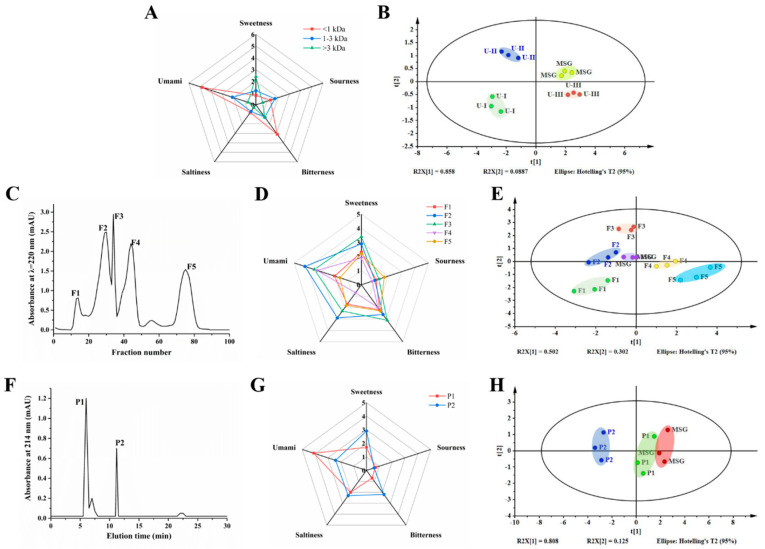
Purification and evaluation of the umami peptides. (**A**,**D**,**G**) Taste characteristic profiles of fractions obtained through ultrafiltration (**A**), gel filtration (**D**), and RP-HPLC (**G**), respectively. (**B**,**E**,**H**) PCA of electronic tongue measurements for fractions obtained through ultrafiltration (**B**), gel filtration (**E**), and RP-HPLC (**H**), respectively. (**C**,**F**) Purification diagram of umami peptides obtained through gel filtration (**C**) and RP-HPLC (**F**).

Ultrafiltration was able to separate crude peptides into fractions of different molecular weight [27]. The hydrolysate was separated into three peptide fractions, including U-I (>3 kDa), U-II (1–3 kDa), and U-III (<1 kDa). As shown in Figure 1A, based on sensory evaluation, the umami taste score rankings of the three fractions were U-III (4.80) > U-II (2.10) > U-I (0.70), with U-III showing the most intense umami taste. Figure 1B presents the PCA plot of ultrafiltration fractions detected by the electronic tongue. The Euclidean distance of the ultrafiltration fractions to MSG was U-I > U-II > U-III. Generally, the shorter Euclidean distance between two samples, the higher the similarity in taste [20]. The nearest fraction to MSG was U-III, suggesting that U-III has the most umami taste. The consistent results in umami taste obtained through sensory evaluation and the electronic tongue were in agreement with those reported by Dang et al. (2019), who found that both evaluation methods presented only a slight difference when they were employed to compare the umami intensity of 36 umami peptides [21]. Thus, our results demonstrated that the umami taste increased with decreasing MW, and that the umami taste of the hydrolysate might be predominant in the U-III fraction. This result was consistent with that reported by Kong et al. (2019), who found that peptide fractions with MW < 1 kDa from *Lentinula edodes* showed the strongest umami taste. Thus, the U-III fraction was freeze dried for further purification [14].

The elution profile of the U-III fraction obtained by the Sephadex G-15 column is shown in Figure 1C. Five eluted peak components, F1- F5, were obtained from the U-III fraction. The separated subfractions were collected for umami evaluation. As shown in Figure 1D, the sensory evaluation rankings of the GFC fractions were F2 > F3 > F4 > F1 > F5. Furthermore, PCA of the electronic tongue showed that that F2 was closest to MSG among the five subfractions (Figure 1E). Our results suggest that the F2 fraction has the strongest umami intensity. Therefore, the F2 fraction was collected and freeze dried for further purification.

The RP-HPLC chromatogram of the F2 fraction is presented in Figure 1F. It was observed that the F2 fraction was separated into three peaks. P1 and P2 with a retention time of 6.0 and 11.2 min, respectively, were the two principal peaks. Thus, the P1 and P2 fractions were collected separately for further umami evaluation. Based on sensory evaluation, the P1 fraction yielded a more intense umami taste than P2 (Figure 1G). The PCA profile (Figure 1H) indicated that P1 was closer to MSG than P2. Our results suggest that the umami peptide of the F2 fraction was predominant in the P1 fraction. In addition, it was evident that P1 was the primary peak in the F2 fraction. Thus, the P1 fraction was screened for further identification.

### 3.2. Identification of Umami Peptides Using LC-MS/MS

LC-MS/MS was employed to determine amino acid sequences of the umami peptides in the P1 fraction. Umami peptide sequences were determined by de novo sequencing and by searching the database. The MS/MS spectrum of peptides is presented in Figure S1. The amino acid sequence and molecular mass of each peptide are shown in Table 1. Five peptides were separated and identified from the *H. marmoreus* hydrolysate: VYPFPGPL, YIHGGS, SGSLGGGSG, SGLAEGSG, and VEAGP, with molecular mass in the range of 471.51 to 889.06 Da and peptide length from 5 to 9. To the best of our knowledge, none of the five umami peptides has been reported on yet, indicating that they were novel umami peptides.

The umami taste characteristics of umami peptides could be influenced by various factors, such as peptide length and structure, and amino acid composition [27,29,30]. In terms of peptide length, the amino acid number of umami peptides usually ranges from 3 to 9, while the molecular mass is less than 1500 Da [17]. Thus, our results on the amino acid number and molecular mass of the five peptides were in agreement with prior research. Aspartic acid (D) and glutamic acid (E) are important umami amino acids [17]. In the present study, none of the five peptides contained D, while two peptides, SG-8 and VP-5, contained the E of 12.5% and 20%, respectively. Liu et al. (2020) found that two of the seven identified umami peptides from *Takifugu rubripes* contained no D or E [31]. Similarly, Yu et al. (2018) found that none of the four identified umami peptides from *Silkworm pupa* hydrolysate contained D or E [32]. Thus, these results suggest that umami amino acid (D and /or E) may not be indispensable in the composition of umami peptides. Moreover, umami-related amino acids, including tyrosine (Y), phenylalanine (F), histidine (H), valine (V), alanine (A), serine (S), glycine (G), and threonine (T), may also contribute to the umami taste [31]. All five identified peptides contained umami-related amino acid, with the proportions in VL-8, YS-6, SG-9, SG-8, and VP-5 being 50%, 83.3%, 88.9%, 75%, and 80%, respectively. Furthermore, although hydrophobic amino acids yielded a bitter taste, many umami peptides were found to contain hydrophobic amino acids, such as leucine (L), isoleucine (I), phenylalanine (F), and proline (P) [13,20]. As reported by Tao et al. (2014), a umami nonapeptide isolated from duck contained six hydrophobic amino acids. Similarly, hydrophobic amino acids were also found in our five peptides [33].

The fundamental requirements for the umami taste formation of long-chain umami peptides were different from those of short-chain peptides [17]. In general, short-chain umami peptides (dipeptides or tripeptides) consisted of D, E, and/or some other hydrophilic amino acids, with the amino acid composition being the most significant factor affecting their umami properties. However, for long-chain peptides, the contents of umami amino acids and hydrophilic amino acids were significantly less than those of short-chain peptides. Even many long-chain umami peptides from various food sources contained no umami amino acids [11]. Furthermore, Bu et al. (2021) found that long-chain peptides had significant spatial effects, and the amino acid composition was no longer the most crucial factor responsible for their activity [27]. Therefore, for the five long-chain peptides identified in this study, it may not be the amino acid composition but the spatial structure, together with the amino acid composition, that formed the basic requirements for their umami taste.

### 3.3. Molecular Docking of Umami Peptides and T1R1/T1R3

**Figure 2:**
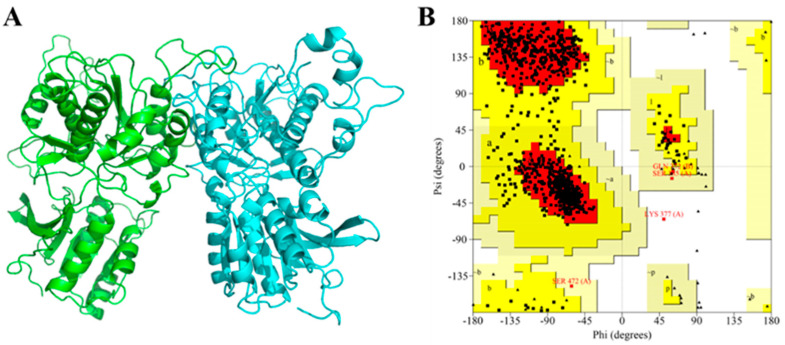
T1R1/T1R3 homology model and reliability evaluation. (**A**) 3D structure of umami receptor T1R1/T1R3 optimized by molecular dynamics simulation. (**B**) Raman map of T1R1/T1R3 homology model.

Molecular docking was employed to investigate the interaction between umami peptides and receptors. Firstly, homology modeling was performed to construct the 3D structure of T1R1/T1R3 due to no crystal structure. The model built with the Swiss-model was optimized using Gromacs, and then evaluated using SAVES [25]. Root-mean-square deviation (RMSD), a critical parameter used to evaluate the stability of simulation systems, can reflect the fluctuation of protein backbones during simulation [34]. The RMSD curve was quickly equilibrated after 5 ns of molecular dynamics simulation (data not shown), indicating that the conformation of T1R1/T1R3 was stable. In this study, in order to obtain an optimal model, 20 ns of molecular dynamics simulation was performed. The final model is shown in Figure 2A. The left subunit was T1R1, while the right subunit was T1R3. In comparison with the original model, the loop region was optimized after molecular dynamics simulation (RMSD value = 3.665). In addition, the Raman map (Figure 2B) shows that 99.9% of residues was in the reasonable region, of which amino acid residues in the most favored regions, additional allowed regions, and generously allowed regions were 84.5%, 15.0%, and 0.4%, respectively. The results demonstrated that the constructed homology model was reasonable [32].

**Figure 3:**
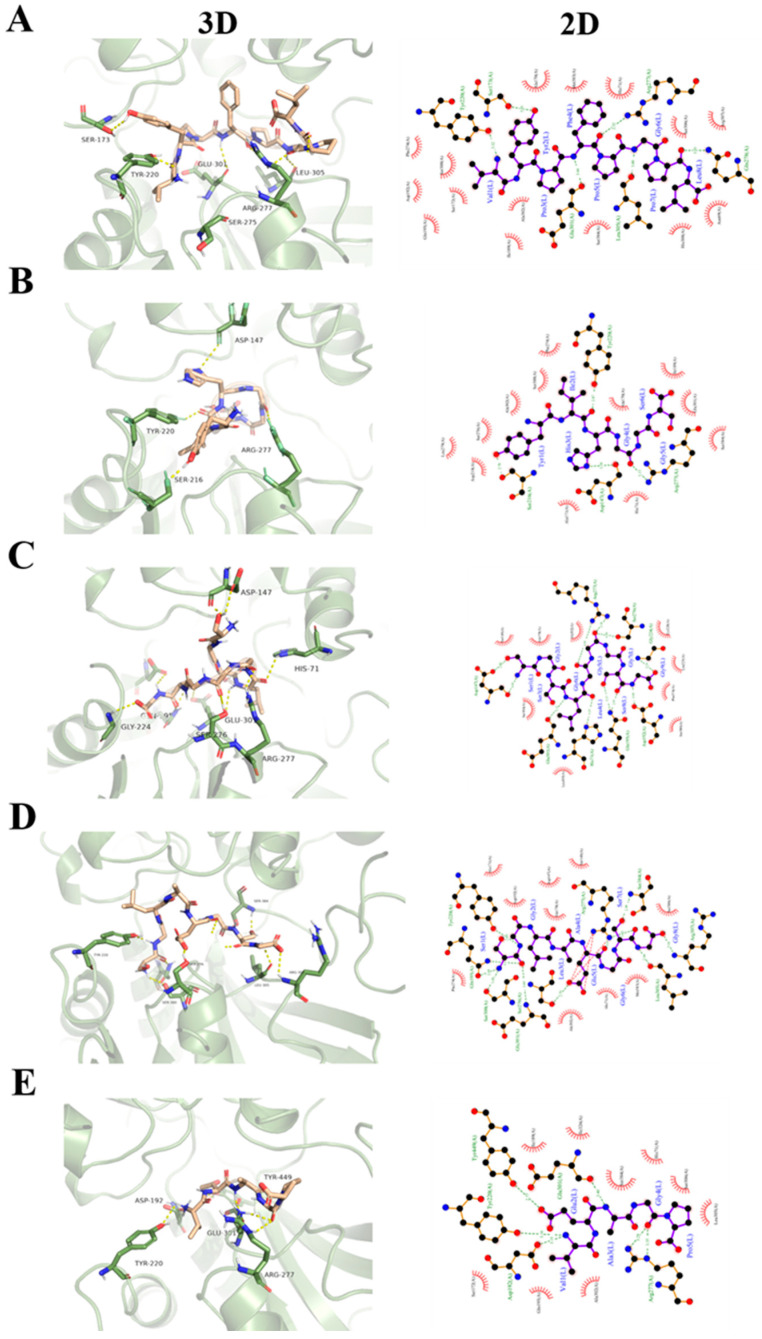
Molecular docking poses of umami peptides with T1R1: (**A**) VL-8, (**B**) YS-6, (**C**) SG-9, (**D**) SG-8, (**E**) VP-5.

Molecular docking was then conducted using the Autodock vina program. Because T1R3 played an auxiliary role in ligand binding, only T1R1 was employed as the molecular docking receptor [35,36]. For each umami peptide, the peptide–T1R1 complex with the highest binding affinity was used as the final docking model. Figure 3 shows the optimal docking poses and the 2D map of the interactions between umami peptides and the active residues of T1R1. It was found that all five peptides could be inserted into the VFTD of a T1R1 subunit. The docking scores of umami peptides with T1R3 are presented in Table 2. The score value reflected the comprehensive effect of the interaction between ligands and receptors. A lower negative score value represented a stronger affinity [13]. The negative score value decreased in the following order: VP-5, SG-9, SG-8, YS-6, and VL-8. The results indicated that VL-8 had the highest affinity for T1R3, followed by YS-6, SG-8, SG-9, and VP-5 (Table 2). 

Hydrogen bonding and hydrophobic interaction played important roles in the binding of the five umami peptides and T1R1. The hydrogen bond numbers of VL-8, YS-6, SG-9, SG-8, and VP-5 binding with T1R1 were 5, 3, 11, 11, and 6, respectively, while hydrogen bond distances ranged from 2.52 to 3.49 Å. This result demonstrates that all five umami peptides can bind to T1R1 with a strong binding force and form a peptide–T1R1/T1R3 complex with stable conformation [13]. Moreover, it was observed that there were 16 residues in the T1R1 subunit involved in the hydrogen bonding with the five peptides, including Arg277, Tyr220, Glu301, Asp147, Asp192, Arg307, Tyr449, Gln195, Gln278, Gly224, Ser173, Ser216, Ser276, Ser300, Ser384, Leu305, and His71. Among these, all five peptides could bind to Arg277, while four peptides could bind to Tyr220 and Glu301. In other words, Arg277, Tyr220, and Glu301 were the most frequently occurring residues in the docking process. This result suggests that Arg277, Tyr220, and Glu301 play an important role in the interaction of the five peptides with T1R1. Our result was similar to that reported by Liu et al. (2019), who also found that Tyr220 and Arg277 were two key residues involved in the binding of a beefy meaty peptide (KGDEESLA) with T1R1 [23]. As reported by Wang et al. (2022), the active sites (D, E, and G) of umami peptides could preferentially bind to T1R1 [24]. We found that at least one E or G in all five peptides formed hydrogen bonds with T1R1, which was in agreement with Wang et al. (2022) [24]. For diverse umami peptides, Arg277 was a highly conserved binding site in T1R1 [24]. It was observed that Arg277 could form hydrogen bonds with G4 in YS-6, G5, and G6 in SG-9, E5 in SG-8, G4 in VP-5, and P5 in VL-8, respectively. Notably, most of the residues belonged to the preferred active sites of ligands. In addition, hydrophobic amino acids had hydrophobic interactions with residues in T1R1. There were 26 residues in T1R1 involved in the hydrophobic interactions. Among these, all five peptides could form hydrophobic interactions with Ala302, while four peptides could form hydrophobic interactions with Ala170, His71, Phe274, and Ser384, respectively. Similarly, Wang et al. (2022) reported that long-chain umami peptides were biased toward residues such as Ala170, Ala302, and Ser384 [24]. Thus, Ala302, Ala170, His71, Phe274, and Ser384 may act as important residues, contributing to the binding of umami peptides with T1R1 through hydrophobic interactions [13,20].

### 3.4. Molecular Dynamics Simulation

Molecular dynamics simulation can reflect detailed information on the ligand and receptor binding process under simulated physiological conditions that help reveal their interaction [37]. However, the influence of umami peptides binding on the dynamics of T1R1 are poorly understood. In this study, the VL-8 with the strongest binding ability to T1R1/T1R3 was selected to investigate their interaction using molecular dynamics simulation. The above VL-8-bound T1R1/T1R3 complex was employed as a starting structure.

**Figure 4:**
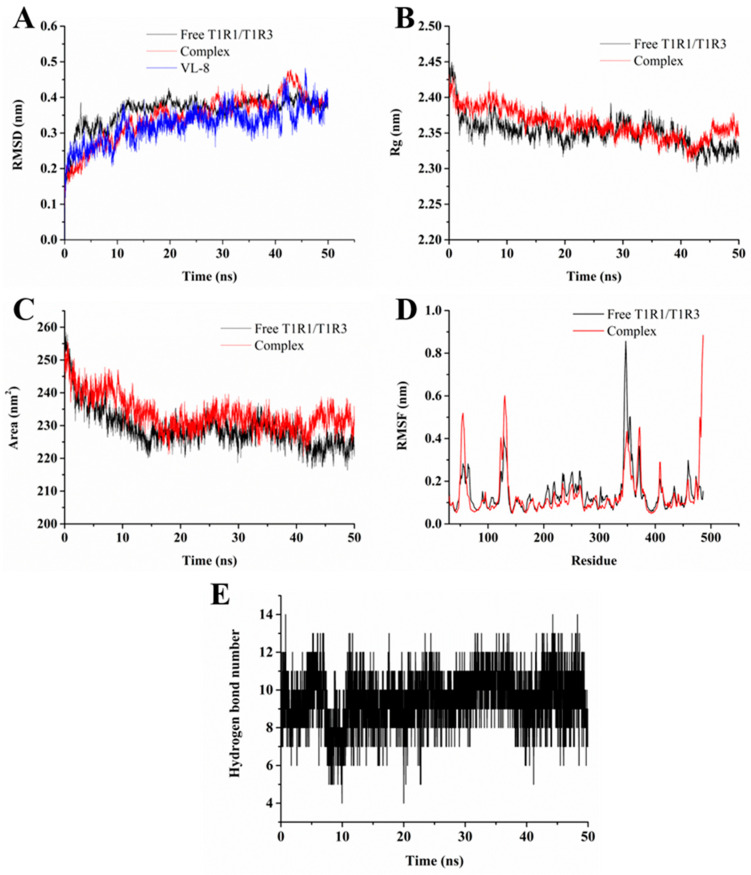
RMSD (**A**), Rg **(B**), SASA (**C**), and RMSF (**D**) plots of free T1R1/T1R3 and VL-8-T1R1/T1R3 complex and hydrogen bond numbers in the complex simulation system (**E**).

As shown in Figure 4A, RMSD values of VL-8 were stable at around 0.34 nm after 10 ns, suggesting that ligands could be steadily packed inside the active pockets of receptors during the 50 ns simulation. RMSD values of T1R1/T1R3 in the presence and absence of VL-8 remained stable at around 0.37 and 0.38 nm after 10 ns, respectively, while the fluctuation was less than 0.2 nm. The results indicated that both simulation systems reached equilibrium [38]. The radius of gyration (Rg) is calculated to evaluate the compactness of a protein. The solvent-accessible surface area (SASA) is an indicator of the contact surface change between the systems and the solvent [26]. The initial Rg values of free T1R1/T1R3 and the complex decreased (Figure 4B). A similar tendency was observed in SASA (Figure 4C). The results indicated that the T1R1/T1R3 structure became more compact. The Rg and SASA values tended to be stable after 10 ns and 15 ns simulation, respectively. The mean Rg values of the complex (2.36 nm) were higher than those of free T1R1/T1R3 (2.35 nm). Similar results were found in SASA (231.62 nm^2^ for complex and 227.17 nm^2^ for T1R1/T1R3). The results suggest that the binding of VL-8 to T1R1/T1R3 induces a looser conformation of T1R1/T1R3. The root mean square fluctuation (RMSF) was employed to investigate the flexibility of individual residues of T1R1/T1R3 [22]. The RMSF values of the complex showed obvious fluctuations in comparison with free T1R1/T1R3 in terms of residues located in Leu61-Ser65, Ala171-Thr175, Ser216-Tyr220, Ser276-Ala280, Glu310-Arg307, Ala369-His373 (Figure 4D). Notably, only the RMSF values of residues in Ala369-His373 significantly increased, while the RMSF values of other residues dramatically decreased. We found that most residues involved in binding from molecular docking also belonged to these flexible residues. Thus, the results indicate that the binding of VL-8 can reduce the fluctuation of the above residues. The hydrogen bond as a key weak interaction helps to maintain binding stability [25]. As shown in Figure 4E, the number of hydrogen bonds in the complex varied in a range of 4 to 14, with 9–10 hydrogen bonds being mostly frequently formed during the simulation. The results indicate that the continuous hydrogen bonds formed between VL-8 and T1R1/T1R3 played an important role in maintaining their binding stability [39].

**Figure 5:**
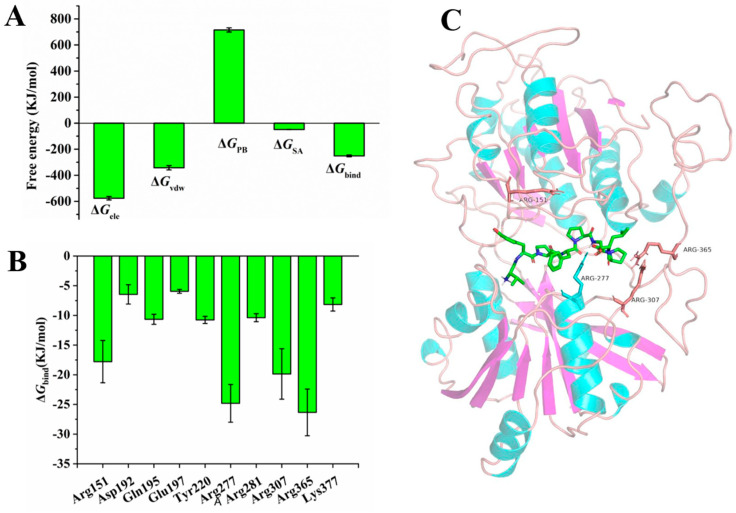
The binding free energy contributions of the VL-8-T1R1/T1R3 complex and positions of key amino acid residues in T1R1. (**A**) Each free energy contribution to total binding free energy. (**B**) Contributions of top ten amino acid residues to binding free energy. (**C**) Positions of Arg residues (151, 277, 307, and 365) in T1R1. The binding mode of VL-8 (shown in green) with T1R1 is derived from the last snapshot of 50 ns simulations.

To quantitatively study the binding affinities of VL-8 with T1R1/T1R3, the Δ*G*_bind_ of the complex was calculated using MM-PBSA [26]. Figure 5A displays the four free energy contributions to total Δ*G*_bind_. The total Δ*G*_bind_ was −251.533 ± 6.818 kJ/mol, indicating that VL-8 could spontaneously bind to receptors. The Δ*G*_ele_ contribution (−576.023 ± 13.028 kJ/mol) was much higher than other energies, demonstrating that electrostatic could be the major driving force in the binding of VL-8 to T1R1/T1R3 [40]. To further determine the per-residue free energy contribution, free energy was decomposed into each individual residue. Ten residues with the lowest binding free energies (accounting for 56.12%) are shown in Figure 5B. The Δ*G*_bind_ of four Arg residues (151, 277, 307, and 365), ranging from −17.788 to −26.338 kJ/mol, were significantly lower than those of other residues (−5.940 to −10.755 kJ/mol), suggesting that these Args could be important contributors to binding affinity [41]. The relative positions of the four Arg residues in the active pockets are shown in Figure 5C. It was evident that VL-8 was sandwiched between Arg151, Arg277, Arg307, and Arg 365. Similar results are reported by Wang et al. (2022) and Liu et al. (2019) [23,24]. Using molecular docking, Wang et al. (2022) found that umami peptides containing more than 7 amino acid residues had a highly remarkable selection to Arg151 and Arg277 [24]. Furthermore, Liu et al. (2019) identified Arg151 and Arg277 as key binding residues using molecular dynamics simulations [23]. In the present study, we explained these phenomena directly by calculating binding free energies. Moreover, from the results of the distinct free energy contributions of the four Arg residues (Table S1), we found that the Δ*G*_ele_ of Arg277 (−122.217 kJ/mol) was much lower than that of other Args (−9.081 to −14.449 kJ/mol). In the previous section, Arg277 was identified as a crucial residue that could interact with all five umami peptides via hydrogen bonding. As mentioned above, Arg 277 was reported to be a highly conserved binding site based on molecular docking [24]. Taken together, we infer that Arg277, as a key binding site, can significantly contribute to the VL-8 binding process via hydrogen bonding.

## 4. Conclusions

Five novel umami peptides were identified from *H. marmoreus*: VYPFPGPL, YIHGGS, SGSLGGGSG, SGLAEGSG, and VEAGP. Molecular docking results demonstrated that all five peptides could be inserted into the active pockets in the VFTD of T1R1. Arg277, Tyr220, and Glu301 in T1R1 may be key binding sites. Hydrogen bonding and hydrophobic interaction were the main interaction forces. In addition, molecular docking scores indicated that VL-8 had the highest affinity for T1R3. Further, molecular dynamics simulations indicated that VL-8 could be stably enclosed inside the binding pocket, while the binding of VL-8 changed the conformation of T1R1/T1R3. The electrostatic interaction could be the main driving force of the VL-8-T1R1/T1R3 complex formation, and four Arg residues (151, 277, 307, and 365) were important contributors to their binding affinities. This study can be used to contribute to the development of umami peptides in edible mushrooms, providing new insights into the taste mechanisms of umami peptides.

## Figures and Tables

**Table 1:** Basic information on the identified peptides.

Abbreviation	Peptide Sequence	*m*/*z*	Amino Acid Number	MW (Da)
VL-8	VYPFPGPL	501.79	8	889.06
YS-6	YIHGGS	632.29	6	632.67
SG-9	SGSLGGGSG	677.30	9	677.67
SG-8	SGLAEGSG	676.30	8	676.68
VP-5	VEAGP	471.23	5	471.51

**Table 2:** Molecular docking scores of umami peptides with T1R1.

Umami Peptide	Score (kcal/mol)
VL-8	−9.143
YS-6	−8.296
SG-9	−6.959
SG-8	−7.723
VP-5	−6.581

## Data Availability

Data is contained within the article or Supplementary Material.

## Supplementary Materials

The following supporting information can be downloaded at: https://www.mdpi.com/article/10.3390/foods12040703/s1, Figure S1: MS/MS spectrum of purified peptides. (A) VYPFPGPL, (B) YIHGGS, (C) SGSLGGGSG, (D) SGLAEGSG, and (E) VEAGP; Table S1: Free energy contributions of four Arg residues.
