# Supplementary material for: Novel Umami Peptides from Hypsizygus marmoreus and Interaction with Umami Receptor T1R1/T1R3
Source: Foods. 2023 Feb 6;12(4):703. doi: 10.3390/foods12040703 (PMC9955199; doi:10.3390/foods12040703)
Supplement: Supplementary file 1 [file foods-12-00703-s001.zip › foods-2128574-supplementary.pdf]

foods

Article

Novel Umami Peptides from *Hypsizygus marmoreus* and Interaction with Umami Receptor T1R1/T1R3

Xiaobo Dong, Chao Wan, Aiyun Huang, Huaide Xu and Hongjie Lei *

College of Food Science and Engineering, Northwest A&F University, Xianyang 712100, China
* Correspondence: leihongjie@nwafu.edu.cn; Tel./Fax: +86-029-87092486

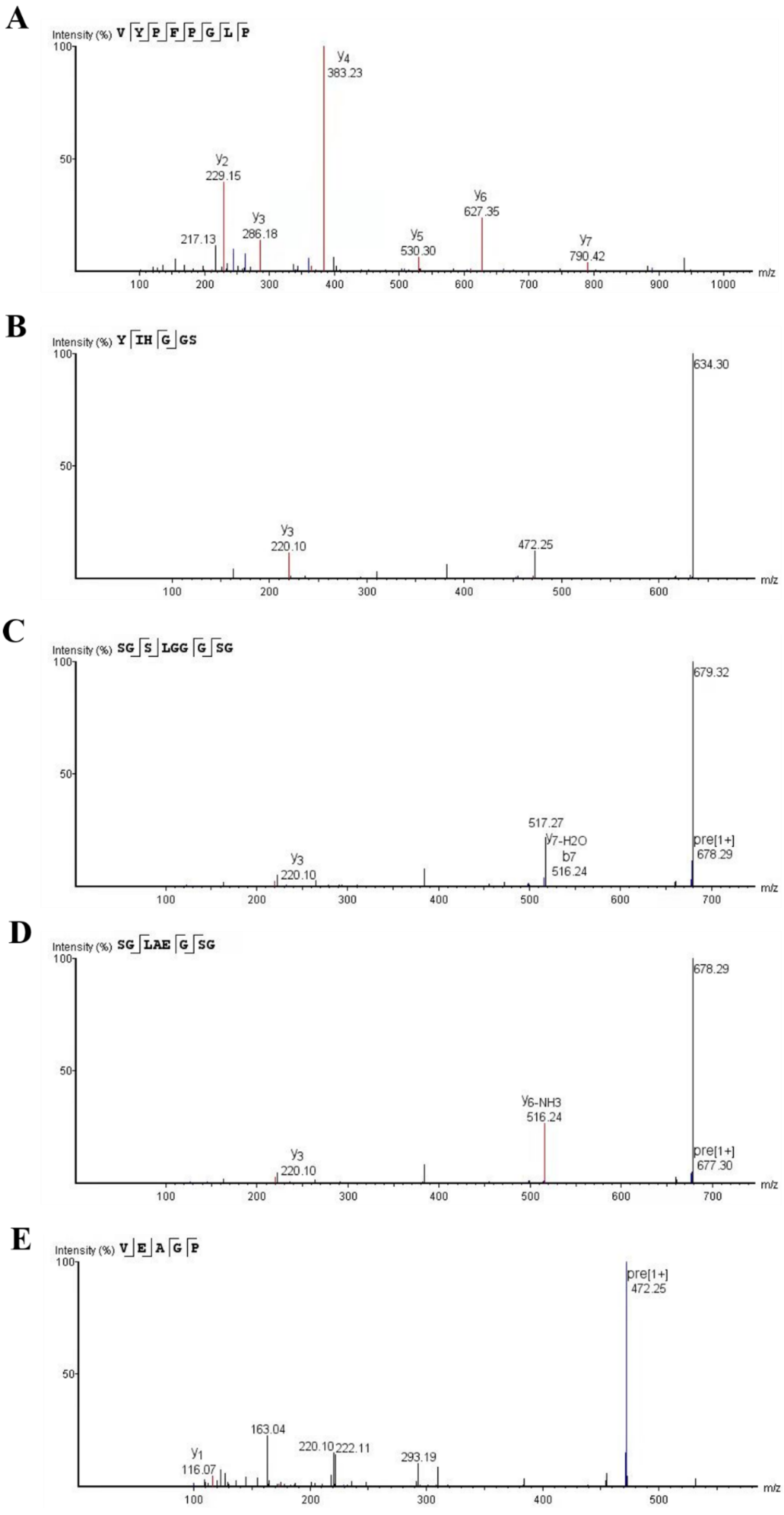

Figure S1. MS/MS spectrum of purified peptides. (A) VYPFPGPL, (B) YIHGGS, (C) SGSLGGGSG, (D) SGLAEGSG, and (E) VEAGP.

Table S1. Free energy contributions of four Arg residues.

**Residue**	**Free energy (KJ/mol)**			
	**Δ*G*ele**	**Δ*G*vdw**	**Δ*G*PB**	**Δ*G*SA**
Arg151	-14.45±3.05	-0.39±0.05	-2.95±0.57	-0.00±0.01
Arg277	-122.22±4.34	-6.16±1.25	106.28±7.99	-2.72±0.05
Arg307	-9.08±0.95	-3.32±2.25	-7.15±1.98	-0.31±0.29
Arg365	-17.31±6.25	-5.91±1.30	-4.03±1.66	-1.01±0.06
